# Analysis of Movement Variability During the Spike Jump Action in Young and High-Level Female Volleyball Players: Differences Between Categories and Playing Positions

**DOI:** 10.3390/jfmk10030268

**Published:** 2025-07-16

**Authors:** Jordi Català, Gerard Moras, Víctor Toro-Román, Carla Pérez-Chirinos Buxadé, Silvia Tuyà-Viñas, Bruno Fernández-Valdés

**Affiliations:** 1 National Institute of Physical Education of Catalonia (INEF), University of Barcelona, 083038 Barcelona, Spain; jcatala@gencat.cat (J.C.); stuyav@tecnocampus.cat (S.T.-V.); 2 Research Group in Technology Applied to High Performance and Health, Department of Health Sciences, TecnoCampus, Universitat Pompeu Fabra, Mataró, 08302 Barcelona, Spain; cperezchirinosb@tecnocampus.cat (C.P.-C.B.); bfernandez-valdes@tecnocampus.cat (B.F.-V.)

**Keywords:** team sport, sport technology, entropy, acceleration, ball

## Abstract

**Objectives**: The aim of this study was to analyze and compare movement variability (MV) during the spike jump (S) action with and without a ball in volleyball players of different categories and playing positions. **Methods**: A total of 48 volleyball players participated in this study. The players were divided according to the following categories: under-14 (U-14) (n = 12); U-16 (n = 12); U-19 (n = 12); and SENIOR (n = 12). Also, they were divided according to playing position: hitters (n = 24); liberos (n = 5); middle blockers (n = 12); and setters (n = 7). The S action with and without a ball was analyzed. Acceleration was analyzed using an IMU device. Acceleration was used to calculate MV through sample entropy (SampEn). **Results**: Differences were observed in all categories when comparing the S action with and without the ball (*p* < 0.001). SampEn was higher in the U-14 category (*p* < 0.001). Regarding playing positions, SampEn was lower in the hitter position compared to the middle blocker (*p* < 0.001) and libero (*p* < 0.001). There were significant inverse correlations between years of experience and SampEn (*p* < 0.05). **Conclusions**: The inclusion of a ball during the S action increases MV. MV is higher in the U-14 category compared to the rest. The hitter position showed lower MV compared to the other playing positions.

## 1. Introduction

Volleyball is an intermittent team sport characterized by short periods of high intensity alternated with rest periods [1,2]. The mean duration of action during a game is 5.99–5.22 s for men’s volleyball players and 7.03–8.72 s for women’s volleyball players, depending on the competition round (quarter-final–final) [3]. Therefore, volleyball is considered an anaerobic sport [4,5]. Specifically, the anaerobic component of volleyball lies in the execution of brief actions such as jumps, blocks, and spikes.

Like other team sports, specialized positions in volleyball allow players to perform different technical and tactical tasks [4]. For example, the mean frequency of block jumps for middles seems to be significantly higher than that for setters and outside hitters [4]. Players develop various skills and execute different technical–tactical tasks during the match [6]. Similarly, biological maturation and age also have a determining influence on sports performance during adolescence [7]. Anthropometric variables (height, body mass, arm span, sitting height, bone diameters, muscle perimeters, and fat, muscle, and bone masses) and physical abilities such as vertical jump and coordination differentiate high-level players [8]. De Alcaraz et al. [9] reported significant reductions in rally and set play times, as well as significant increases in the number of jumps per set comparing under-14, under-16, under-19, national, and international categories.

Volleyball differs from most team sports because a net divides the court, and the ball can only be hit, never carried. Consequently, the execution of movements is conditioned by the need to clear the net and deliver the ball to the other side of the court [10]. Among volleyball skills, the spike (S) is a complete technical action that includes several phases: the approach, take-off, optional ball contact, and landing [11]. Points scored by S account for more than 70% of points in competitions [12] and have the highest correlation with the final match outcome [13,14]. Successful execution of S involves great complexity and is crucial for performance [15]. The need to combine vertical jump ability with a prior run and a final hit in suspension increases the difficulty of the action, making it highly demanding in terms of coordination [11]. Sex- and experience-related differences in S have been reported. Specifically, male players apply a higher approach speed and higher upper body inclination compared to female players [11]. On the other hand, experienced volleyball players landed with a large ankle dorsiflexion range of motion compared to novices. In addition, they jumped significantly higher than novices [16]. These findings suggest that both biomechanical and neuromuscular factors contribute to performance differences, which could be further understood by analyzing the variability of movement patterns.

Variability is an inherent characteristic of nature observed in biological systems [17]. Movement variability (MV) is described as the set of habitual variations that occur during the execution of multiple repetitions of the same task [18,19,20]. The analysis of human movement has evolved to assess the variability of a measure, focusing on detecting changes in fluctuations and the spatiotemporal characteristics of the results. Linear analyses of human movement have several recognized limitations, mainly in determining the degree of complexity and the time-dependent structure of a time series [21,22]. These limitations can be complemented by using nonlinear analyses, such as entropy measures. The advantages of this method lie in the additional information it provides about the relationships between levels of a biological system and the organization of an athlete’s movement from a dynamic systems perspective [23,24]. Currently, the most widely used methods for biological data are approximate entropy (ApEn) and, more recently, sample entropy (SampEn) and multiscale entropy (MSE) [23,25,26]. 

The analysis of MV in sports has traditionally been approached through linear methods. However, with the development of nonlinear analysis algorithms [25] and advancements in measurement technologies, particularly wearable technology such as inertial measurement unit (IMU) devices, nonlinear methods have become increasingly used. These methods have been applied to the study of physiological parameters [27,28], as well as gait and running patterns [29,30,31]. They have also been employed to analyze movement in sports involving striking with implements [32,33,34,35], snow sports [36] and team sports [22,24,32,37,38].

In volleyball, few studies have employed nonlinear analysis. Existing research primarily focuses on analyzing the tactical variability indices of players [39]. However, MV in volleyball-specific technical actions has not been extensively studied [40], and even fewer studies have compared MV across different categories and playing positions. Regarding differences between categories, previous studies suggest that MV may decrease with practice [41] and experience [42]. Additionally, MV has been observed to vary depending on the playing position [24,37], as well as the inclusion of an implement or ball [43,44]. Given this, the aim of the present study was to analyze and compare MV during the S action, both with and without a ball, among volleyball players from different categories and playing positions. Based on this, we hypothesize that MV during volleyball actions may differ depending on the category and playing position. Specifically, MV would be lower in higher categories and in playing positions where the S action is predominant.

## 2. Materials and Methods

### 2.1. Participants

A total of 48 female volleyball players from a top-level national club participated in this study. The participants were divided into categories—U-14 (n = 12); U-16 (n = 12); U-19 (n = 12); and SENIOR (n = 12)—based on the classification by the Royal Spanish Volleyball Federation (RFEVB) (Table 1). The SENIOR team played in the Superliga Femenina 2 (Spain). Additionally, the participants were divided based on their playing position: hitters (n = 24); liberos (n = 5); middle blockers (n = 12); and setters (n = 7) (Table 2). All participants trained a minimum of 3 sessions per week, each lasting at least two hours.

After verbally explaining the objectives and procedures of this study, written informed consent was obtained from the participants’ parents. The procedures complied with the Declaration of Helsinki (2013) and were approved by Comitè d’ètica d’investigacions clíniques de l’Administració esportiva de Catalunya (03/2015/CEICEGC).

The inclusion criteria for this study were as follows: (i) no injuries preventing jumping or hitting the ball; (ii) regular training for the past 30 days; (iii) at least 2 years of experience as a federated volleyball player; (iv) at least 2 years of experience in the assigned playing position; and (v) no use of medication or supplements during the study. The exclusion criteria were as follows: (i) any illness or injury that could hinder optimal execution of the S; (ii) belonging to a category lower than U-12; and (iii) less than 3 years of experience as a federated volleyball player.

Age and experience were collected via a questionnaire before the study began. Anthropometric measurements (height and weight) were taken during the first evaluation using a scale with a stadiometer (IC6003; Medinox, Zaragoza, Spain). For the measurements, participants stood upright against a vertical wall, ensuring their back, buttocks, and heels were in contact with the wall. Their head was positioned in the Frankfort plane, and the stadiometer was placed lightly on the top of their head without applying pressure.

### 2.2. Study Design

**Figure 1:**
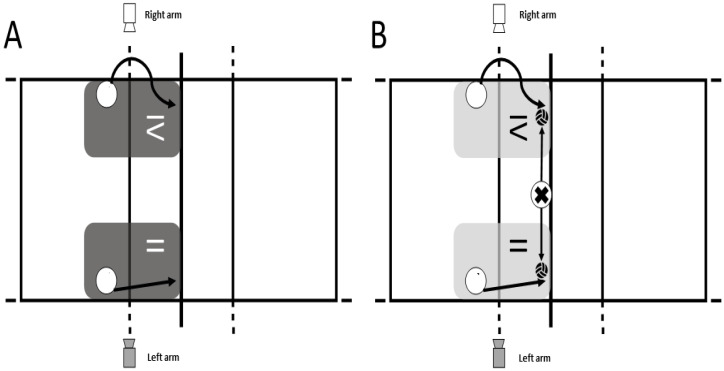
Study design. (**A**): run-up and S without a ball in zones II and IV; (**B**): run-up and S with a ball in zones II and IV; white circle: participants performing the action; “x” circle: setter.

A randomized crossover study was conducted. S executions, with and without a ball, were performed in zone II (right) and zone IV (left) of the court [45] (Figure 1). The study lasted for two weeks (14 days) and consisted of three sessions. In the first session (day 1), the participants’ sports and anthropometric data were collected. During the second session (days 4–7), the players performed the S action in four randomized sets, either with or without a ball, from zones II and IV. In the final session (days 11–14), the players completed the remaining four sets from the second session. In both the second and third sessions, each player performed 4 sets of 6 repetitions, with and without a ball (a total of 48 S per player).

### 2.3. Assessment

The assessments were performed before the training sessions to avoid fatigue. The sessions were performed on the same day across different weeks. Before the second and third sessions, the players performed an 8 min warm-up divided into the following stages: (i) general joint mobility; (ii) continuous running; (iii) changes in direction (lateral and anteroposterior); (iv) vertical and horizontal jumps; and (v) hitting the ball without jumps near a wall using the dominant arm. The intensity of the hitting progressively increased. The warm-up concluded with a set of 4 S at the net, performed on both the right and left sides of the court (zones II and IV, respectively). The net height was set according to the official regulations for each category (U-14: 2.10 m; U-16: 2.18 m; U-19 and SENIOR: 2.24 m).

After the warm-up, the type of S (with or without a ball) and the zone of execution (zone II or zone IV) were randomized. Each player performed the S action with the net at the official height according to their category, using their dominant arm. Two sets of 6 repetitions of S were performed for each condition in random order: (i) 12 repetitions in zone IV without a ball; (ii) 12 repetitions in zone II without a ball; (iii) 12 repetitions in zone IV with a ball; and (iv) 12 repetitions in zone II with a ball. In total, each player executed 48 S across the two sessions. After each repetition, there was a 10 s pause, and between sets, there was a 3 min pause to limit the effects of fatigue on jumping capacity [46].

For executions with a ball, an expert setter performed the setup using a ball that he threw himself. The expert setter performed the attack at third tempo (the attacker waits for the ball to ascend and then performs a three-step or longer approach). S actions were only recorded if the setup allowed for a comfortable hit and the ball landed within the boundaries of the opposing court. The setup was executed from the center of the net toward the net antenna, with a height of at least 2 m above the top of the net.

During S execution, no specific instructions were given to the players. If the S action did not clear the net or went out of bounds, the action was repeated until each set had 6 recorded jumps.

### 2.4. Equipment

This research was conducted on a volleyball court with official measurements, approved for participation in national competitions, equipped with a set of poles and a net authorized by the Royal Spanish Volleyball Federation (RFEVB), with the height adjusted according to the category. All actions were performed using a specific volleyball ball (Molten V5M5000, Hiroshima, Japan).

All sessions were recorded using a high-speed camera (Casio Exilim EX-ZR100, Tokio, Japan) to capture every action (210 fps). The camera was positioned on a tripod (Hama Star 63 166-3D, Monheim, Germany), with its focal axis perpendicular to the trajectory of the S. The camera was placed on the opposite side of the player’s hitting arm (Figure 1). The camera was used to synchronize the IMU signal with the video of each session for additional visual verification.

After warming up, the players wore a specially designed rigid belt to hold an IMU device. Two different sizes were used to ensure the belt fit snugly against the body, with the most appropriate size selected for each player. The belt did not restrict the players’ movements during the S execution. The belt and IMU compartment were designed to ensure the device remained fully attached to the body, preventing unwanted movements and oscillations [47]. The device was placed on the player’s lower back, near the body’s center of mass, at L5 [22,48].

**Figure 2:**
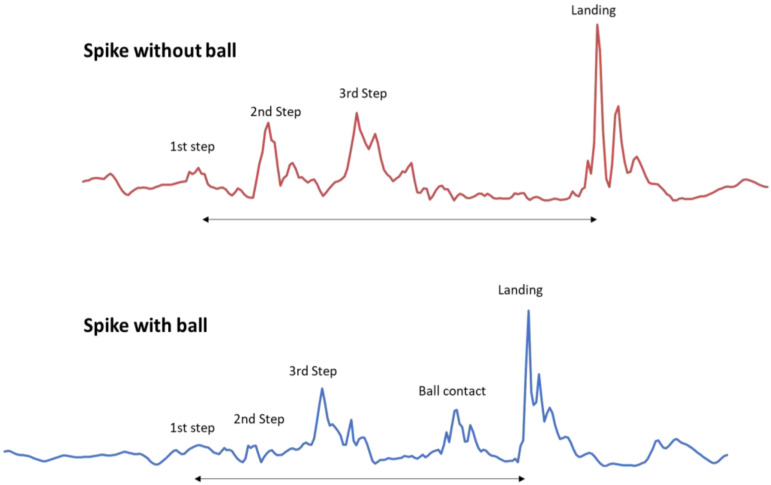
Acceleration signals from a spike performed with and without the ball.

One of the methods for measuring MV is the calculation of entropy based on acceleration data collected using IMUs [49]. This has been addressed in previous studies [22,24,37,50]. In the present study, the acceleration of volleyball players was measured using an IMU device (WIMU, Realtrack Systems, Almeria, Spain; weight: 70 g; size: 81 mm × 45 mm × 15 mm). The IMU also contains a 3D magnetometer recording at 100 Hz. Signals from the 3-axial accelerometer (range: ±400 G; sampling frequency: 1000 Hz) were used. For data analysis, SPRO software (version 987, Realtrack Systems, Almeria, Spain) was used to obtain the raw signal and perform an initial visual analysis to verify its clarity (Figure 2). The WIMU (Realtrack Systems, Almeria, Spain) inertial device is considered a useful tool for measuring the vertical jumping ability of athletes [51,52,53]. Total acceleration was calculated using the vector sum in three dimensions: vertical (z), anteroposterior (y), and mediolateral (x) (1).

(1) $$Total\;acceleration=\sqrt{z^{2}+y^{2}+x^{2}}$$

### 2.5. Data Analysis

Acceleration was used as the main parameter, while distance and velocity were discarded for the following reasons: (i) The IMU device measures acceleration (direct measurement), whereas velocity and displacement are obtained through indirect calculations from the acceleration signal [54]. (ii) In addition, to calculate velocity, acceleration must be integrated with respect to time using mathematical and computational techniques that must include corrections for noise affecting the signal structure. To facilitate the subsequent synchronization of the acceleration signal with the video, the WIMU was tapped by hand in front of the camcorder before and after each series of finishes to generate an acceleration peak that was easily detectable in the acceleration signal, allowing subsequent synchronization with the start and end of the video.

Cuts were applied to the acceleration signal for each repetition of the S from the approach run phase to the landing phase. The phases of which the S is composed are as follows [11]: (i) approach; plant (ground contact prior to take-off); (ii) take-off; flight (incorporating the movements of the body prior to ball contact); (iii) the hitting action; (iv) landing; and (v) recovery. These signals were exported to Excel, where sample entropy (SampEn) was calculated for each signal, according to Richman and Moorman [55] using custom routines programmed in Matlab^®^ (MATLAB 9.13 R2022b, The MathWorks, Natick, MA, USA) to analyze the MV for each action. The appropriate values for the parameters—sequence length (m), tolerance (r), and data length (N)—for calculating SampEn depend on the type of data and the context of the analysis. In the present study, m = 2 offers a good balance between sensitivity and analytical capability in short recordings [55]. Also, r = 0.2 × SD (standard deviation) was selected, as this value is widely used in standard studies, balancing sensitivity and robustness to noise [55]. Finally, N was set greater than 1000 data points, as recommended for the selected m and r values [56]. The formula for calculating the SampEn is detailed below [55].

(1) We formed a vector m, X (1) to X (N − m + 1), defined as follows:
  (2) $$X\left(i\right)=\left[x\left(i\right),x\left(i+1\right),\ldots ,X\left(i+m-1\right)\right]\;i=1,N-m+1$$
(2) Define for each *I*, for *i* = 1, *N* − *m*
  (3) $$B_{i}^{m}\left(r\right)=\frac{1}{N-m+1}\times no.of\;d_{m}\left[X\left(i\right),X\left(j\right)\right]\leq r,\;i\neq j$$
(3) Similarly, define for each *I*, for *i* = 1, *N* − *m*
  (4) $$A_{i}^{m}\left(r\right)=\frac{1}{N-m+1}\times no.of\;d_{m+1}\left[X\left(i\right),X\left(j\right)\right]\leq r,\;i\neq j$$
(4) Then define the following:
  (5) $$B^{m}\left(r\right)=\frac{1}{N-m}\overset{N-m}{\underset{i=1}{\sum }}B_{i}^{m}\left(r\right)$$
  (6) $$A^{m}\left(r\right)=\frac{1}{N-m}\overset{N-m}{\underset{i=1}{\sum }}A_{i}^{m}\left(r\right)$$
(5) Finally, we calculate *SampEn*:
  (7) $$SampEn\left(m,r,N\right)=-ln\left(\frac{A^{m}\left(r\right)}{B^{m}\left(r\right)}\right)$$

The entire signal was divided into small homogeneous segments corresponding to each of the S performed with and without a ball, and heterogeneous parts were removed. The length of the segments (m = 2) was found to be sufficient to ensure the reliability of SampEn. The signal was sub-sampled from 1000 Hz to 400 Hz, and it was found that in both cases, the main dynamics were captured.

### 2.6. Statistical Analysis

Statistical analyses were performed using IBM^®^ SPSS^®^ Statistics v2. Figures were created using the R software (v4.1.2, The R Foundation for Statistical Computing, Vienna, Austria) and GraphPad Software 8 Inc. (Boston, MA, USA). For all S, the average SampEn of the executions performed in zones II and IV, both with and without the ball, was analyzed. A total of 384 actions were analyzed (48 participants × the mean of the 6 repetitions of each set (2 sets) in either of the 4 conditions of S [zone IV without the ball; zone IV with the ball; zone II without the ball; zone II with the ball]).

Normality of the sample was assessed using the Kolmogorov–Smirnov test. Levene tests were used to assess the normality and homogeneity of variances. A two-way ANOVA (ball effect and category/playing position effect) was used to assess differences in the studied variables. The Bonferroni post hoc test was used to determine differences between categories and playing positions. The effect size was calculated using partial eta squared (ηp^2^), where 0.01–0.06, 0.06–0.14, and >0.14 were considered small, moderate, and large effect sizes, respectively [57]. Regression lines were also fitted between the average of all SampEn values from the S (n = 48) and years of experience. Pearson’s correlation coefficient (r) and the coefficient of determination (R^2^) were analyzed. Values of *p* < 0.05 and *p* < 0.01 were considered significant and highly significant differences, respectively [57].

The relationship between SampEn and years of experience was also analyzed using the cluster method. First, an elbow test was conducted to determine the number of clusters. The elbow method is a graphical technique used to determine the optimal number of clusters in a dataset. It is based on plotting the total variation explained by the model against the number of clusters. This variation is typically represented as a function of the number of clusters, and a point where the reduction in explained variation begins to decrease significantly, forming an “elbow” in the graph, is observed. This point suggests the optimal number of clusters for analysis [58]. Once the optimal number of clusters was identified using the elbow method, a more detailed analysis was performed using the K-means clustering algorithm. This algorithm is widely used in unsupervised data analysis to group similar observations into clusters based on the similarity of features [59].

## 3. Results

**Figure 3:**
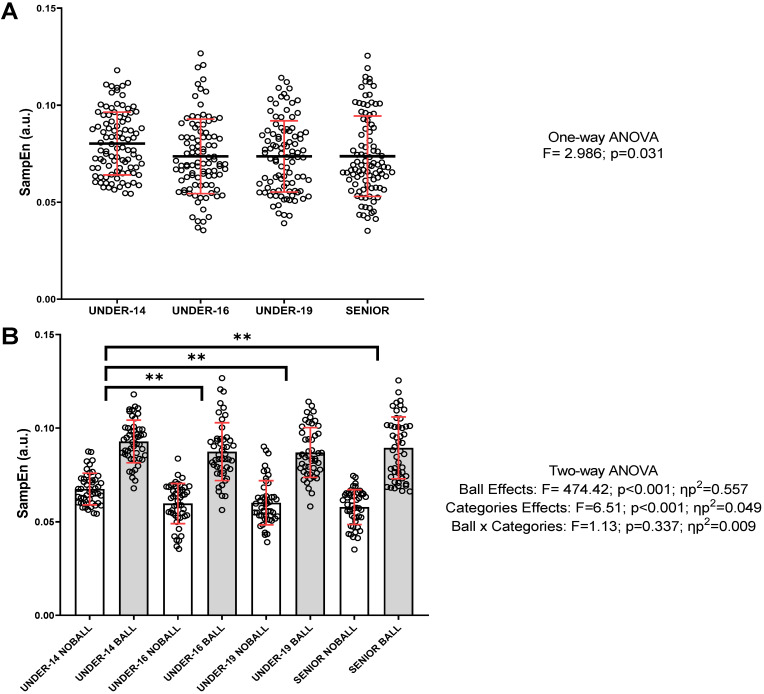
General values and the influence of the ball during the S action on the entropy values (SampEn) for each category. (**A**) The general SampEn values (with and without the ball) divided by category. (**B**) SampEn values for the S with and without the ball for each category. Individual values, means, and standard deviations are shown. In (**A**) The mean is represented by a black line and the standard deviation by a red line. Statistical significance: ** *p* < 0.01; ηp^2^: partial eta squared.

Figure 3 illustrates the SampEn values for the different categories. Figure 3A represents the overall SampEn values (with and without the ball), categorized by group. Significant differences were observed between groups (F = 2.986; *p* = 0.031). However, Bonferroni’s post hoc test did not reveal any significant differences (U-14 vs. U-16, *p* = 0.088; U-14 vs. U-19, *p* = 0.087; U-14 vs. SENIOR, *p* = 0.095). Moreover, Figure 3B displays significant differences across all categories when comparing the S performed with and without the ball (F = 472.42; *p* < 0.001; ηp^2^ = 0.557), with higher SampEn values when the action was executed with the ball. Regarding differences between categories, significant differences were also found (F = 6.51; *p* < 0.001; ηp^2^ = 0.049), specifically between U-14 and the other categories (U-16, U-19, and SENIOR = *p* < 0.01). There was no significant interaction between factors.

**Figure 4:**
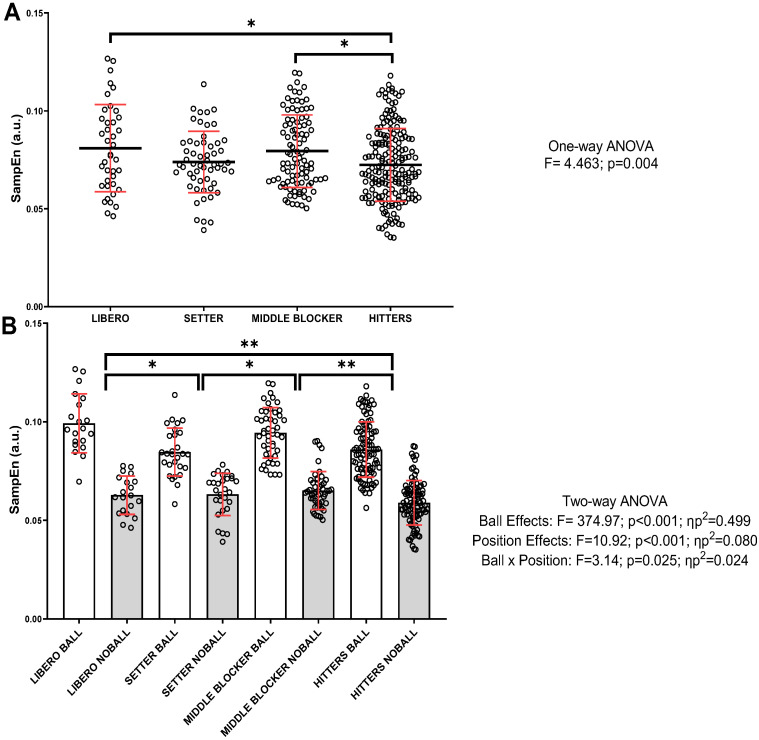
Global entropy values (SanpEn) and the influence of the ball during S action for each playing position. (**A**) General SampEn values (with and without the ball) separated by category. (**B**) SampEn values according to the category and S action with and without the ball. Data are expressed as individual values, mean, and standard deviation. In (**A**), the mean is shown with a thick black bar and the standard deviation in red. In (**B**), the bar represents the mean values, and the standard deviation is shown in red. * *p* < 0.05 ** *p* < 0.01; ηp^2^: partial eta squared.

Figure 4 presents the SampEn values for each playing position. Figure 4A shows the global SampEn values for the S performed with and without the ball. Significant differences were found between playing positions when the global action was analyzed (F = 4.463; *p* = 0.004), specifically between hitters and liberos, as well as hitters and middle blockers (*p* < 0.05). Figure 4B shows the SampEn values for each playing position separately, comparing the S action with and without the ball. A positive interaction was found between the factors playing position and ball (F = 3.14; *p* = 0.025; ηp^2^ = 0.024). Significant differences between all playing positions were observed when comparing S with and without the ball (F = 374.97; *p* < 0.001; ηp^2^ = 0.499), with higher values when the ball was involved. In terms of position comparisons, significant differences were also found (F = 10.92; *p* < 0.001; ηp^2^ = 0.080). Specifically, differences were found between hitters vs. middle blockers (*p* < 0.001; vs. liberos, *p* < 0.001) and setters (vs. middle blockers, *p* = 0.043; vs. liberos, *p* = 0.032), with lower SampEn values in the last-mentioned positions.

**Figure 5:**
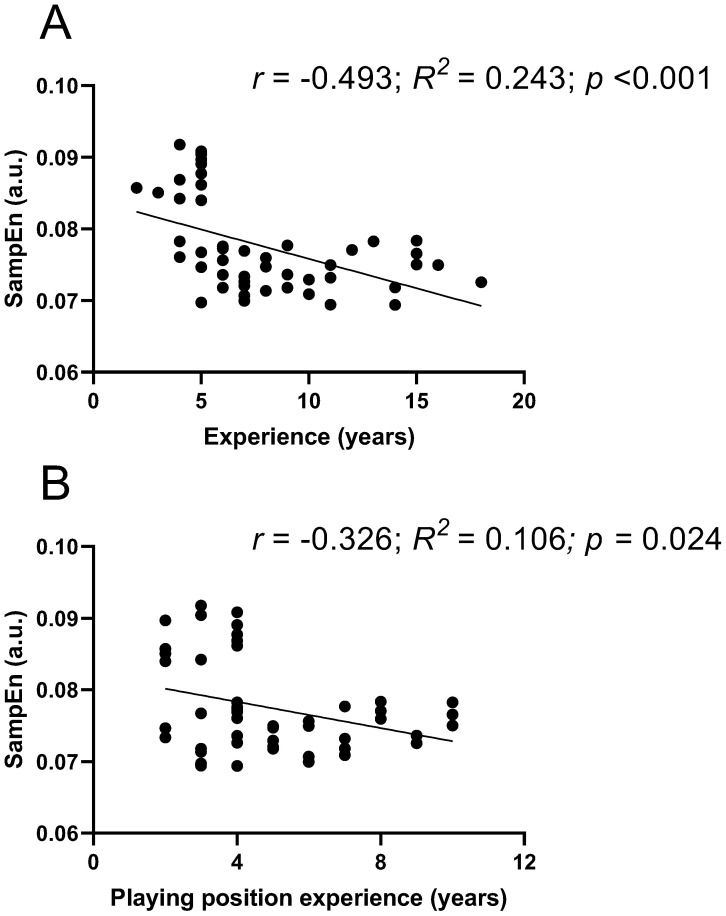
Correlation between years of experience playing volleyball and all SampEn values obtained during S actions under the different analyzed conditions. (**A**) Regression line between SampEn values and years of experience playing volleyball. (**B**) Regression line between SampEn values and years of experience in the volleyball playing position; r: Pearson correlation coefficient; R^2^: coefficient of determination.

Figure 5 shows the regression lines between years of experience playing volleyball and years of experience in the playing position, with the average SampEn values obtained across all strikes (average of the 48 executed strikes). Significant correlations were observed both with total experience (r= −0.493; R^2^ = 0.243; *p* < 0.001) and with years of experience in the playing position (r= −0.326; R^2^ = 0.106; *p* = 0.024).

**Figure 6:**
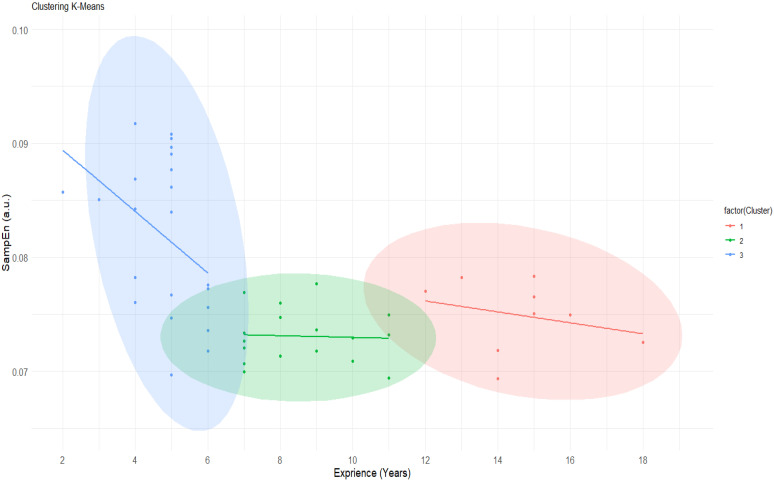
K-means cluster analysis between SampEn and total years of experience playing volleyball. SampEn: sample entropy.

Finally, Figure 6 shows the cluster analysis. The elbow method determined that the present data should be defined by three clusters to be analyzed using the K-means clustering method. In Figure 6, three clearly defined groups can be observed, where a first group (blue) shows high levels of entropy (SampEn), a second group (green) is in the intermediate years of experience where SampEn values stabilize, and a final group (red) includes players with more years of experience and SampEn values similar to those in the green group.

## 4. Discussion

The aim of the present study was to analyze and compare MV during the S action, with and without the ball, among volleyball players from different categories and playing positions. It was observed that MV was higher in the U-14 category compared to the other categories. On the other hand, MV was higher in the middle blocker and libero playing positions. Similarly, total years of experience and years of experience in a specific playing position were inversely correlated with SampEn.

In offensive play, S is the most effective attack move associated with match success [60]. Due to the specificity and complexity of S in volleyball, it is essential not only to possess strength and power but also technical and coordination aspects that contribute to performance [60].

MV evaluates movement regularity, which is a recognized indicator of motor control [61,62]. It also provides valuable information about the player’s coordination characteristics and sheds light on the task’s dynamics [63]. It was expected that MV would be lower in expert players due to their greater ability to control the coordination of joint space degrees of freedom through regular practice [64,65,66]. The fact that this reduction and stabilization of MV already appears at the U-16 stage may be due to the fact that the movement pattern for S matures at this stage and remains relatively stable in subsequent categories. It is known that, between the ages of 10–12 in girls, a peak height velocity occurs, which can result in changes in height of ~8 cm/year. Growth-related changes may be associated with a temporary reduction in motor coordination [67]. A motor pattern like S develops through three distinct stages: initial, elementary, and mature. As each pattern progresses through these stages, clear and notable changes in body actions occur [68]. The maturity of a pattern is related to a formal reference model and is independent of age [69]. The analyzed motor pattern seems to improve throughout each stage. It is worth noting that the critical period or golden age of motor skill development/learning is between the ages of 6–12, and most authors agree that the onset of puberty marks the end of this period [70]. Within the critical period, skills are acquired faster compared to later developmental stages [70].

Previously, Serrien et al. [71] analyzed the S with 3D kinematics in professional players and junior players. The authors reported that there were no differences in pelvis and trunk orientation angles when comparing young players with adult players. However, they found significant differences between young and professional players in terms of the speed reached by these body parts during the three phases of the S. In turn, Serrien et al. [71] also described changes between young and adult players in elements of the aerial phase of the S. Specifically, they found a higher lateral tilt speed of the trunk at the moment of impact and a lower height at contact due to greater elbow flexion during the arm’s movement toward the ball in junior players. All these elements likely generate minimal variations in the movement pattern, even though they are key determinants of precision and accuracy in the action [72,73]. According to these results, it is possible that when players reach the maturity of their movement pattern, they generate a unique performance profile [33]. In relation to the above, it has been reported that the MV of an action decreases as it is trained [50]. The training process appears to regulate the stability and adaptability of movement to the extent that the motor system adapts to perturbations [74].

Regarding the results found in this study, it is possible that from the U-16 category onwards, the consistency of the internal and external environment is similar, which could explain the few variations in MV. Movements should not show random variability produced simply by noise but compensatory and adaptive variability, a consequence of the continuous adjustment to the environment [75]. Athletes’ movements occur by combining, coordinating, and coupling the variabilities that exist at multiple levels of the internal and external environments [75]. Not only will greater reproducibility of the S reduce MV, through increased consistency in the internal environment, but the external environment will also tend to be more consistent when performing the motor skill [75]. As players progress from novices to experts, they acquire a greater perceptual capacity that allows them to improve and optimize motor patterns, which results in this more consistent external environment. It is possible that the greater the experience, the better the control of information acquisition mechanisms, leading to longer and more precise fixations [76]. They also adapt better to anomalous ball trajectories [77,78] and are able to multitask better while performing a motor task [79]. This has been demonstrated in the present study through regression analysis, where an inverse relationship is observed between MV and sports experience, as well as in the cluster analysis. The cluster analysis also shows three distinct groups. The first group (blue) obtains higher SampEn values and the sharpest decline in these values with years of experience. On the other hand, in groups 2 (green) and 3 (red), with more years of experience, SampEn values decrease and stabilize, possibly related to greater motor control of the S action.

Furthermore, the inclusion of the ball during the S action increased MV in all participants, regardless of category and position. Previous studies have reported this in rugby players [22,50], basketball players [43], and hockey players [44]. This could demonstrate that the inclusion of the ball during the S action demands higher levels of coordination patterns, stimulating the beneficial and adaptive aspects of variability in the system’s function [74]. During the S action with the ball, the central nervous system modulates the anticipatory and compensatory activities of distal and proximal muscles differently than in the action without the ball [22]. It should be noted that a ball machine was not used in order to analyze a real response to the game action. It is important to note that all sets were performed by the same setter.

Regarding the differences between positions, the present study observed lower MV in hitters. The study design might have influenced these results. In this study, a high set was chosen for both attack zones (zone II and zone IV), which is the type of set these players normally face, whereas middle blockers face lower sets, mainly in the central area of the court [80]. On the other hand, setters and liberos do not usually perform the S action, which could explain why SampEn might be higher. Considering that athletes manage to reduce the MV of their sports movements through practice [81], it was expected that in this motor pattern with the ball, placed high in zone II and zone IV, the lowest MV would appear in the hitters, as they accumulate the most repetitions in these positions throughout a season [82] and throughout their sports career. Studies on the change in movement complexity with practice find that, in general, complexity could be reduced through practice [83,84]. In a study conducted on rugby players analyzing MV changes in tackles for different positions, it was observed that forwards had the lowest MV of all players [24], with practice and experience in that movement being essential to reducing MV [85]. Similarly, differences in MV by position were also observed in soccer players, with attackers having higher MV in a decision-making task involving passing. In volleyball, hitters perform around 40,000 S in a season [73,86], and the lower MV values obtained in our study could suggest that hitters adjust better to performing the S with the ball. This reduction could be due to an improved ability to control the coordination of ball passing during practice [42]. Based on the optimization principle, sensory estimation could minimize uncertainty through optimal integration and minimize variability in motor production through optimal control [87].

### Limitations

The present study is not without its limitations: (i) Only females were evaluated. (ii) It was an isolated action far from game reality. (iii) Only two zones (zone II and zone IV) of the court were used. (iv) The findings in the present study should not be generalized, as male players were not tested in this study. Anthropometric and physiological differences between the sexes could influence the action of S. (v) The setter was the same expert player and there could be variability in the action S. However, a setter was used to make the actions as similar as possible to the real game. Future studies could analyze differences between sexes and playing positions, as well as the execution of S in different areas of the court, such as zone III.

## 5. Conclusions

In conclusion, MV during the execution of the S is higher in the U-14 category compared to the other categories (SUB-16, SUB-19, and SENIOR).

The hitter position showed lower MV compared to the other positions (middle blocker, libero, and setter).

The inclusion of a ball during technical action increases the player’s coordination demands, reflected in an increase in MV. This could be of interest for introducing new stimuli.

Finally, MV is inversely correlated with the years of volleyball practice experience and years of experience in the playing position.

## Figures and Tables

**Table 1:** Participant characteristics by category.

Category	Age (Years)	Height (m)	Weight (kg)	Experience (Years)
U-14 (n = 12)	13.83 ± 0.39	1.73 ± 0.05	57.18 ± 5.74	4.33 ± 0.98
U-16 (n = 12)	15.67 ± 0.49	1.77 ± 0.04	65.06 ± 3.69	6.08 ± 1.24
U-19 (n = 12)	18.08 ± 0.79	1.79 ± 0.05	69.90 ± 6.40	8.58 ± 2.39
SENIOR (n = 12)	22.67 ± 2.53	1.81 ± 0.05	73.99 ± 6.15	12.92 ± 3.42

U-14: under-14; U-16: under-16; U-19: under-19.

**Table 2:** Participant characteristics by playing position.

Category	Age (Years)	Height (m)	Weight (kg)	Experience (Years)	Position Experience (Years)
Hitters (n = 24)	16.83 ± 3.62	1.76 ± 0.06	65.95 ± 7.15	7.50 ± 3.73	4.92 ± 2.28
Liberos (n = 5)	19.80 ± 3.37	1.71 ± 0.04	63.88 ± 5.39	11.40 ± 3.61	4.80 ± 2.40
Middle blockers (n = 12)	18.42 ± 3.40	1.82 ± 0.03	72.57 ± 6.39	7.17 ± 3.54	5.08 ± 2.61
Setters (n = 7)	17.00 ± 3.51	1.77 ± 0.02	68.67 ± 5.03	8.57 ± 4.50	4.86 ± 2.19

## Data Availability

The original contributions presented in the study are included in the article. Further inquiries can be directed to the corresponding author.

## Abbreviations

The following abbreviations are used in this manuscript:

ApEn	Approximate entropy
IMU	Inertial measurement unit
MV	Movement variability
MSE	Multiscale entropy
RFEVB	Royal Spanish Volleyball Federation
SampEn	Sample entropy
S	Spike jump
U-14	Under-14
U-16	Under-16
U-19	Under-19
